# Analytical Determination of Cephalosporin Antibiotics Using Coordination Polymer Based on Cobalt Terephthalate as a Sorbent

**DOI:** 10.3390/polym15030548

**Published:** 2023-01-20

**Authors:** Maria A. Chernomorova, Marina S. Myakinina, Vladimir A. Zhinzhilo, Igor E. Uflyand

**Affiliations:** Department of Chemistry, Southern Federal University, Rostov-on-Don 344090, Russia

**Keywords:** coordination polymers, cephalosporin antibiotics, adsorption, solid-state extraction, sorbents

## Abstract

In this work, a coordination polymer based on cobalt terephthalate was obtained and characterized by elemental analysis, infrared spectroscopy, X-ray diffraction analysis, and scanning electron microscopy. The coordination polymer was tested as a sorbent for the solid-phase extraction of cephalosporin antibiotics, including ceftriaxone, cefotaxime, and cefazolin, from aqueous solutions. The coordination polymer had a high adsorption capacity (520.0 mg/g). Antibiotics adsorption followed pseudo-second order kinetic model and the Freundlich isotherm model. The calculated thermodynamic parameters indicate a spontaneous process. The resulting coordination polymer has good stability and reusability. The possibility of separating the studied cephalosporins on a chromatographic column filled with a coordination polymer was shown. This work opens great prospects for the development and application of a coordination polymer based on cobalt terephthalate for the removal of cephalosporins from ambient water.

## 1. Introduction

**Figure 1:**
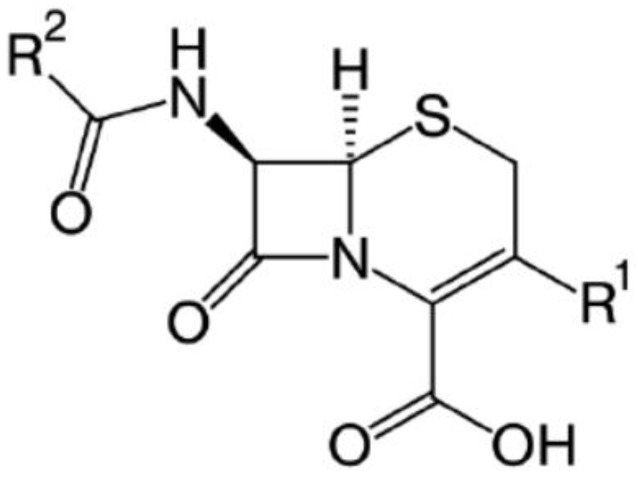
Structural formula of cephalosporins.

Cephalosporins belong to the class of β-lactam antibiotics. They can reveal similarities in structure and mechanism of action with other β-lactam antibiotics, such as penicillins, carbapenems, and monobactams (Figure 1) [1,2,3].

Cephalosporins belong to a group of antibiotics with a diverse spectrum of action. They inhibit the synthesis of the peptidoglycan layer of the cell wall [4]. Currently, cephalosporin antibiotics are widely used in medicine, animal husbandry and agriculture. They treat infectious diseases and are used as nutrients that help livestock grow [5]. Due to the wide and strong spectrum of bactericidal action of cephalosporins, their production and use in clinical practice is constantly growing. Global production of cephalosporin antibiotics was USD 13.69 billion in 2019 and will reach USD 16.87 billion by 2027 [6]. The most common uses of cephalosporins are aquaculture and human and veterinary medicine [7,8,9,10,11]. Incorrect use of cephalosporin antibiotics for the treatment of disease or growth promotion in livestock results in significant drug residues in dairy products. This problem poses a significant risk to consumer health and technology challenges for the dairy industry [12,13].

As many studies have shown [14,15], the presence of these substances is increasing in the surrounding world. Many antibiotics without metabolism enter the aquatic environment with excrement. This fact poses a huge threat to human health and the environment. Due to the active use of cephalosporin antibiotics, they have been found in various aquatic environments, which can lead to environmental and health problems. The detection of cephalosporin antibiotics in aquatic environments raises great concern about their potential impact on the environment and human health. That is why the problem of pollution of water bodies due to the excessive use of antibiotics seems to be especially important [16]. The concentration of some antibiotics in natural and urban water can even reach the level of mg/L [17]. The analytical determination of cephalosporins and their residues in the environment is significant since they favor the emergence of resistant bacterial strains.

To date, various methods have been developed for the removal of cephalosporin antibiotics from their aqueous solutions. Most of the methods are associated with technologies such as biological [18,19,20] and membrane technologies [21], as well as the oxidation processes [22,23,24]. Of greatest interest is the analysis of cephalosporins both in pure form and in pharmaceuticals and biological fluids [1,25]. Today, chromatographic techniques are widely used for the analysis of cephalosporins, including high-performance liquid chromatography (HPLC) [26,27,28,29,30,31], ultra-high performance liquid chromatography (UPLC) [14], high-performance thin layer chromatography [32], and capillary electrophoresis (CE) [33,34,35,36].

The adsorption method is a promising method for the removal of antibiotics, as it has several advantages, in particular, ease of operation, greater removal efficiency and the reuseability of the adsorbent [37,38,39,40,41,42]. Among the disadvantages of traditional adsorbents, one should note the low adsorption capacity, the difficulty of separation, and the lack of individual adjustment [43]. Therefore, an important task is the development of efficient and economical sorbents for the adsorption of antibiotics from natural and wastewaters. Their distinguishing characteristics should include high adsorption capacity, high adsorption rate, and ease of separation.

In connection with the task, the attention of researchers was attracted by coordination polymers (CPs), which consist of clusters or ions of metals and organic polytopic ligands, and are characterized by a large specific surface area, diverse crystal structure, and good porosity [44,45,46,47,48]. By adjusting the structure and functionality of CPs, as well as by post-synthetic modification, one can easily change their physico-chemical characteristics for the improvement of adsorption performance. The unique characteristics of CPs have allowed them to be used to effectively remove various contaminants, including antibiotics, from natural and wastewater [49,50,51,52,53,54]. As an example, we note the adsorption of antibiotics by CP based on terephthalic acid and 1,10-phenanthroline [55,56,57].

Among the various CPs, MOF-71 can be considered one of the best studied and readily available Co-containing CPs based on 1,4-benzenedicarboxylic acid (H_2_BDC) [58]. The architecture of this CP includes infinite chains of corner-sharing CoO_6_ octahedra and BDC linkers connect each strand to four parallel strands [59].

The aim of this work was to find environmentally friendly synthetic methods that are convenient to use and cause minimal environmental damage. As a rule, the syntheses of such compounds are carried out with prolonged heating, which is associated with significant energy consumption and the use of hazardous solvents, for example, DMF, benzene [58]. We used a two-step procedure with the isolation of Co(OH)_2_ to obtain CP followed by its interaction with H_2_BDC. CP was characterized by various analytical methods: X-ray powder diffraction, elemental analysis, and scanning electron microscopy. In addition, the resulting CP was used to remove cephalosporin antibiotics from their aqueous solutions.

## 2. Materials and Methods

### 2.1. Starting Materials

Ethanol (EtOH, 98%), ethyl acetate, methylene chloride, dimethylformamide (DMF), chloroform, dichloromethane, cobalt(II) nitrate hexahydrate (Co(NO_3_)_2_·6H_2_O, ≥99.0%) and 1,4-benzenedicarboxylic acid (95.0%) were supplied by Sigma-Aldrich and were used without prior purification.

### 2.2. Antibiotics

Cephalosporin antibiotics obtained from Sigma-Aldrich were chosen as sorbates: cefotaxime, ceftriaxone, cefazolin (Table 1).

The initial solution containing cephalosporin had a concentration of 100 mg/L. Working solutions were prepared by dissolving their weighed portions and dilution in deionized water obtained using Millipore Simplicity water purification system (Merk Millipore, Burlington, MA, USA).

### 2.3. Synthesis of Coordination Polymer

Synthesis of cobalt terephthalate was carried out at relatively low temperatures and minimal procedures for purification of the target product without the use of process modulators. The synthetic technique consisted of a two-stage approach. At the first stage, 5.82 g (0.01 mol) Co(NO_3_)_2_·6H_2_O was dissolved in 20 mL of water and a solution containing 1.6 g (0.02 mol) NaOH in 20 mL of freshly boiled water was added. The Co(OH)_2_ precipitate was separated by centrifugation, washed several times with hot water until no nitrate ions remained in the washing water (by reaction with diphenylamine). The resulting compound was transferred as a suspension to a flask, 3.32 g (0.01 mol) H_2_BDC was added and refluxed for 30 min (until the lilac-violet color turned pink), separated by filtration under vacuum, and washed first with hot water, and then hot DMF. An amount of 4.90 g of a pink crystalline powder were obtained, corresponding to a yield of 89% based on cobalt terephthalate dihydrate. Elemental analysis: found (%): C, 36.4; H, 2.86; Co, 22.5. Calculated for [Co(H_2_O)_2_(BDC)] (%): C, 37.1; H, 3.00; Co, 22.1. To use the resulting complex as a sorbent, it was conditioned (activated). To do this, a sample of cobalt terephthalate was heated in a dynamic vacuum at a temperature of 150 °C for 8 h.

### 2.4. Characterization

Elemental analysis was performed using a CHNOS vario EL cube analyzer (Elementar Analysensysteme GmbH, Langenselbold, Germany). Cobalt was determined on an energy dispersive X-ray fluorescence spectrometer «X-Art M» (Comita, Moscow, Russian) or atomic absorption spectrometer «MGA-915» (Lumex, St. Petersburg, Russia). The Fourier transform IR (FTIR) spectra were recorded with a Nicolet 380 FTIR spectrometer (Thermo Fisher Scientific, Waltham, MA, USA) from KBr pellets using Softspectra data analysis software (Shelton, CT, USA). X-ray diffraction (XRD) analysis was carried out on a DRON-UM-2 diffractometer (JSC “Burevestnik”, St. Petersburg, Russia) with CuKα radiation (λ_Cu_ = 1.54184 Å) in the range of 2θ = 5–80° angles 2θ with a scanning speed of 5°/min and a temperature of 25 °C to determine the phase composition and crystallite size. Scanning electron microscopic (SEM) images were obtained using a Zeiss LEO SUPRA 25 device (Carl Zeiss, Jena, Germany) at an accelerating voltage of 3 kV.

### 2.5. Experiments on Equilibrium Adsorption of Antibiotics

The batch method has been applied to equilibrium studies because of its ease of use. Solutions of antibiotics with a volume of 40 mL and a concentration of 20, 10, 5, 2.5 and 1.25 mg/L were placed in a 100 mL beaker and thermostated at 278, 293, and 308 K on a magnetic stirrer. After the specified temperature was reached, 10 mg of the sorbent was added to each solution. 10 mL of the sorbent suspension was taken from each antibiotic solution after 3 h and subjected to centrifugation for 5 min. A UV-visible spectrophotometer (Varian, Cary 50, Palo Alto, CA, USA) was used to determine the residual antibiotic concentration.

The following equations were used to calculate the amount of antibiotic adsorbed:

(1) $$q_{t}=\frac{(C_{0}-C_{t})V}{m}$$

(2) $$q_{e}=\frac{(C_{0}-C_{e})V}{m}$$

where q_t_ and q_e_ are the amounts (mg/g) of antibiotics adsorbed on the sorbent at time t and in the equilibrium; C_0_, C_t_, and C_e_ are the concentrations of antibiotics in the solution (mg/L) at the initial stage, at time t, and in the state of equilibrium; m and V represent the amount of sorbent (g) and the volume of antibiotic solution (L).

The following equation was used to calculate the degree of adsorption R (%):

(3) $$R\;\left(\%\right)=\frac{\left(C_{0}-C_{e}\right)}{C_{0}}*100\%$$

### 2.6. Adsorption Experiments

The Langmuir isotherm model assumes adsorption on separate adsorption centers with the formation of a monomolecular adsorption layer, while the centers are energetically equivalent, and the sorbed particles do not interact with each other or their interactions are insignificant [62]. The Langmuir adsorption isotherm equation in linearized form has the following form [63]:

(4) $$\frac{C_{e}}{q_{e}}=\frac{1}{K_{L}\;q_{m}}+\frac{C_{e}}{q_{m}}$$

where K_L_ is the Langmuir constant (L/mg) related to the affinity of the sorbent and sorbate binding sites. This value indirectly characterizes the free energy of sorption; q_m_ is a value representing the maximum adsorption capacity. At this moment, the active areas of the sorbent surface are completely covered with the molecules of the object under study; this parameter can be used to compare the adsorption characteristics of different sorbents (mg/g).

The dimensionless equilibrium parameter R_L_, used to confirm the favorable adsorption process, was calculated using the following equation:

(5) $$R_{L}=\frac{1}{1+K_{L}C_{0}}$$

The nature of adsorption can be irreversible (R_L_ = 0), favorable (0 < R_L_ < 1), linear (R_L_ = 1), or unfavorable (R_L_ > 1) [64].

The Freundlich adsorption isotherm relates the equilibrium amount of the adsorbed antibiotics on a sorbent (q_e_) to the equilibrium concentration C_e_ of the antibiotics in solution on a heterogeneous adsorbent surface [65]. Its linear form is expressed as:

(6) $$\ln q_{e}=\ln K_{F\;}+\frac{1}{n}\ln C_{e}$$

where 1/n is a constant showing the increase in the amount of adsorbed substance, which is proportional to the root of the n-th degree of the concentration of the solution; in addition, this constant can serve as a parameter of the heterogeneity of the sorbent surface in a comparative analysis of sorbents, K_F_ is the Freundlich constant.

The value of the parameter 1/n indicates the irreversible (1/n = 0), favorable (0 < 1/n < 1), or unfavorable (1/n > 1) nature of adsorption [1].

### 2.7. Study of Adsorption Kinetics

In accordance with the pseudo-first-order kinetic model [66], the filling rate of active sorption sites on the sorbent is proportional to the number of unoccupied sites and is calculated using the following equation:

ln(q_e_ − q_t_) = lnq_e_ − k_1_t (7)

In accordance with the pseudo-second order kinetic model [67], adsorption includes the interaction between the adsorbate and two independent unoccupied sites of the adsorbent [68]. This model in its linear form is described by the following equation:

(8) $$q_{t}=\frac{t}{\frac{1}{k_{2}q_{e}^{2\;}}+\frac{t}{q_{e}}}$$

where k_2_ is pseudo-second order adsorption rate constant (g/mmol min).

The thermodynamic parameters make it possible to evaluate the direction, feasibility, and possibility of adsorption. The standard free energy (∆G^0^), standard enthalpy (∆H^0^), standard entropy (∆S^0^) and adsorption activation energy (E_a_) were calculated using the following equations:

(9) $$K_{D}=\frac{q_{e}}{C_{e}}$$

(10) $$∆G^{0}=-\mathrm{RT}\ln K_{D}$$

(11) $$\ln K_{D}=\frac{∆G^{0}}{R}-\frac{∆H^{0}}{\mathrm{RT}}$$

where K_D_ is the adsorbate distribution coefficient.

The parameters ∆H^0^ and ∆S^0^ were calculated from the slope and intersection of the plot ln K_D_ versus 1/T, respectively.

### 2.8. An Experiment on The Separation of Antibiotics in a Chromatographic Column

For this experiment, a small layer of sealing material was placed in columns 1.7 cm in diameter and 15 cm long, then filled with conditioned cobalt terephthalate, periodically tapping the bottom of the columns on the surface of the table to compact the sorbent. With a layer length of 12 cm, 20 mL of the antibiotic solution under study was poured into each column. Elution was carried out after 15 min. A mixture of methanol and hydrochloric acid (4:1) was used as the eluent. The determination of the antibiotic in the eluate was carried out using a qualitative drop reaction. It was found that the beginning of the elution of cephalosporin antibiotics occurred after passing 3–4 mL of a blank eluent. An amount of 10 mL of eluate containing antibiotics was collected. The concentration of cefotaxime, ceftriaxone and cefazolin was determined spectrophotometrically. The experiment was repeated three times. To separate a mixture of antibiotics, a column was prepared as described above, and filled with a mixture of three antibiotics with initial concentrations of 25 mg/L, and 20 mL of the mixture was added to the column. Elution was carried out with a mixture of methanol and hydrochloric acid (50:1 by volume).

## 3. Results

### 3.1. Synthesis and Identification of Cobalt Terephthalate

**Figure 2:**
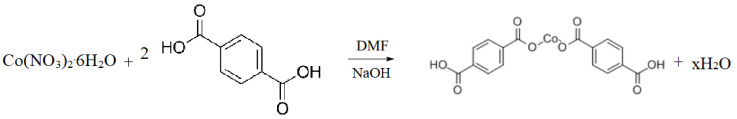
Overall reaction for the synthesis of cobalt terephthalate.

**Figure 3:**
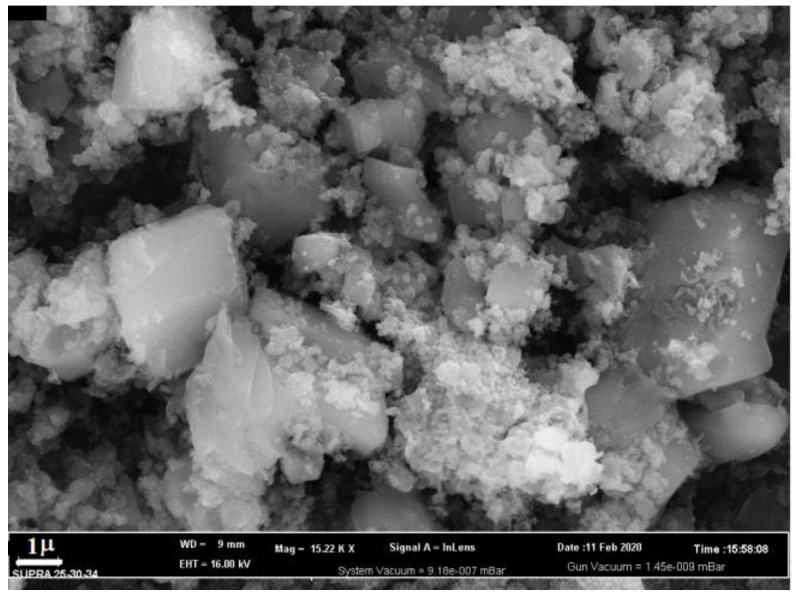
SEM image of cobalt terephthalate crystals.

Cobalt terephthalate was synthesized at low temperatures without the use of process modulators. The synthetic technique consisted of two stages. At the first stage, cobalt hydroxide precipitate was formed during the interaction of cobalt salt and alkali, and at the second stage, cobalt terephthalate was obtained because of the reaction of H_2_BDC and cobalt hydroxide. The overall reaction is shown below (Figure 2):

The crystal morphology was determined using SEM (Figure 3). It can be seen from the figure that the crystals have a cubic shape with dimensions of 2 × 2 × 2 μm and a monolithic structure.

**Figure 4:**
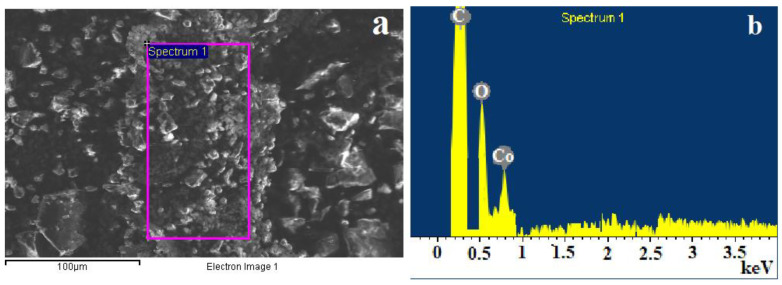
EDX analysis of a sample of cobalt terephthalate: (**a**) analyzed area, (**b**) EDX spectrum.

Energy dispersive analysis (EDX) data confirm the elemental composition of the resulting cobalt terephthalate (Figure 4).

**Figure 5:**
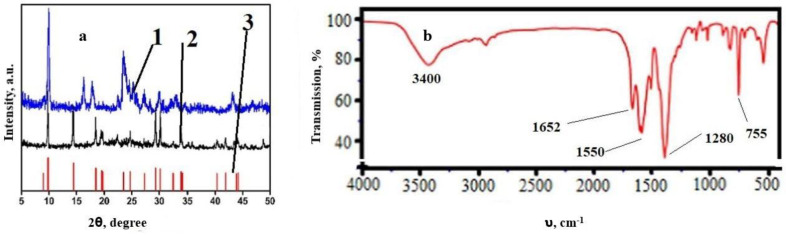
XRD (**a**) and IR spectrum (**b**) of cobalt terephthalate. 1—Cobalt terephthalate dihydrate, 2—sample dried in a vacuum with heating, 3—peaks of the XRD profile stored in the international database (JCPDF 34-1896).

The identification of the obtained compound was carried out using XRD and IR spectroscopy. X-ray diffraction analysis showed a good agreement between the main peaks of cobalt terephthalate with those previously published and the database (Figure 5a). Satisfactory coincidence of the peaks indicates the phase purity of the resulting compound.

The IR spectrum of the obtained compound (Figure 5b) showed a broad, but relatively weak, band at 3400 cm^−1^, associated with the vibrations of hydroxo groups of water, as well as an intense absorption band at 1280 cm^−1^, related to asymmetric vibrations of hydroxo groups, 1550 cm^−1^, associated with symmetrical vibrations of hydroxo groups and at 755 cm^−1^, characteristic of the Me–O bond, which proves the formation of cobalt–oxygen bonds in the sorbent. Since there is no signal in the spectrum in the region of 1720 cm^−1^, it can be argued that all carboxyl groups are deprotonated, and the target product was obtained. Overall, the obtained values coincide with those previously published [69].

### 3.2. Solid Phase Extraction of Cephalosporin Antibiotics

**Figure 6:**
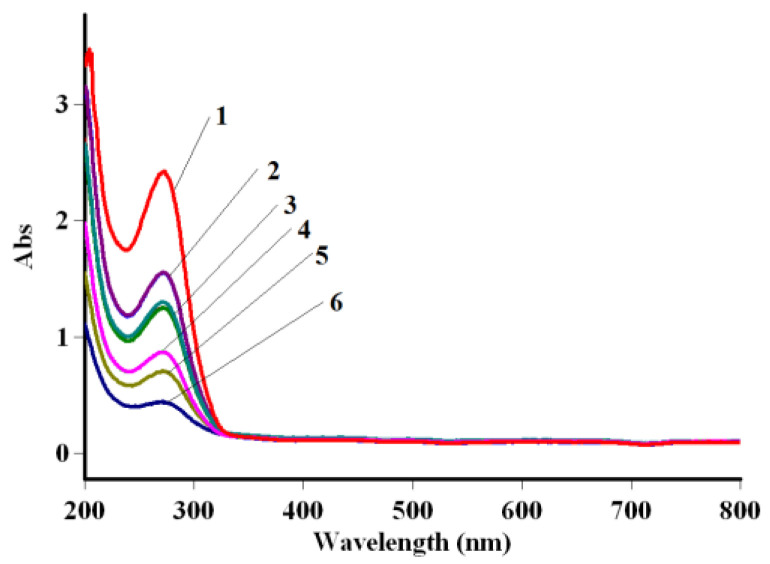
UV-visible absorption spectra of CTX antibiotic before (1) and after extraction for 5 (2), 10 (3), 15 (4), 30 (5) and 45 (6) min.

The ability of the synthesized cobalt terephthalate to adsorb cephalosporin antibiotics from aqueous media was studied with their subsequent UV detection (Figure 6).

**Figure 7:**
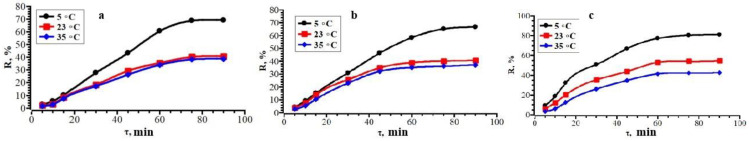
The dependence of the degree of extraction on the contact time for CZO (**a**), CTX (**b**) and CTX (**c**).

The dependence of the degree of adsorption on time at different temperatures is shown in Figure 7. When comparing the data of the three graphs, it can be seen that the degree of adsorption for each antibiotic is higher at a lower temperature. For ceftriaxone, the degree of adsorption is higher than for cefazolin and cefotaxime at room temperature.

**Figure 8:**
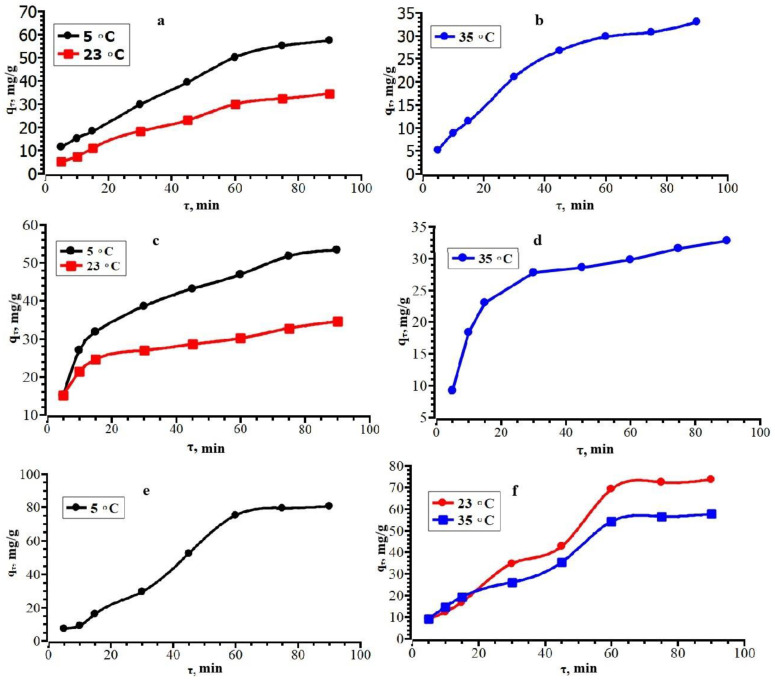
Kinetic curves of solid phase extraction versus contact time for CZO (**a**,**b**), CTX (**c**,**d**), and CRO (**e**,**f**).

Most of the sorbate is absorbed in the first 60 min, followed by gradual leveling off and reaching a plateau after 75–90 min (Figure 8). This may be since at the beginning there are many absorbing centers that are filled during the reaction. It can be concluded that equilibrium occurs 1.5 h after the start of the process.

**Figure 9:**
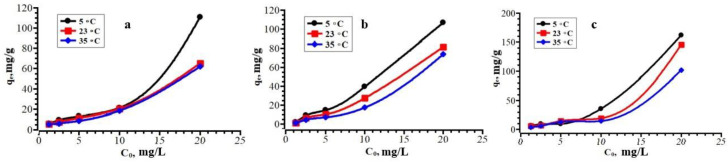
Dependence of the value of solid phase extraction on the initial concentration of CZO (**a**), CTX (**b**) and CRO (**c**).

The dependences of the of sorption of antibiotics on their initial concentration are shown in Figure 9. It follows from the data obtained that the sorption capacity decreases with a decrease in the initial concentration of the sorbate. The graphs show that when antibiotics reach concentrations of 50–100 mg/L, there is a sharp increase in the degree of adsorption. This pattern may indicate that at the first stages the sorbate concentration is too low, and the number of active sorption centers is maximum, and with an increase in the amount of sorbate, their sharp decrease occurs. These graphs show the possibility of absorbing a larger amount of antibiotic that exceeds the maximum permissible concentration (MPC) norms (100 mg/L). At the same time, there is a tendency to increase the sorption capacity with decreasing temperature.

**Figure 10:**
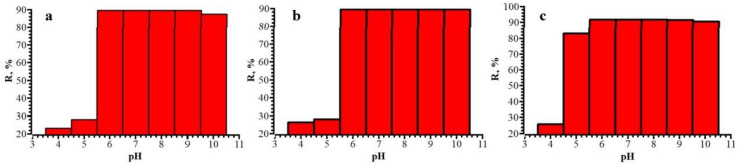
Dependence of the degree of extraction on the pH of the medium for CZO (**a**), CTX (**b**), CRO (**c**).

One of the most key factors influencing the adsorption process is pH since it determines the nature of the interaction between the adsorbent and antibiotics. The influence of pH on antibiotics adsorption was studied in the range from 3.5 to 10.5. The dependence of the degree of adsorption on pH of the medium is shown in Figure 10. According to the data obtained, the degree of adsorption increases in a neutral and alkaline medium and is lower in the case of an acidic medium.

Summing up the above dependencies, we can conclude about the optimal parameters for the adsorption of antibiotics of the cephalosporin series by cobalt terephthalate. The initial concentration can vary from 6.125 to 100 mg/L, but antibiotics with the highest concentrations showed the best results in terms of sorption capacity. Kinetic studies show that equilibrium occurs within 1.5 h after the introduction of the sorbate. It has been established that low temperatures are the most favorable for sorption. Suitable values for the acidity of the environment are in the range from neutral to strongly alkaline. 

**Figure 11:**
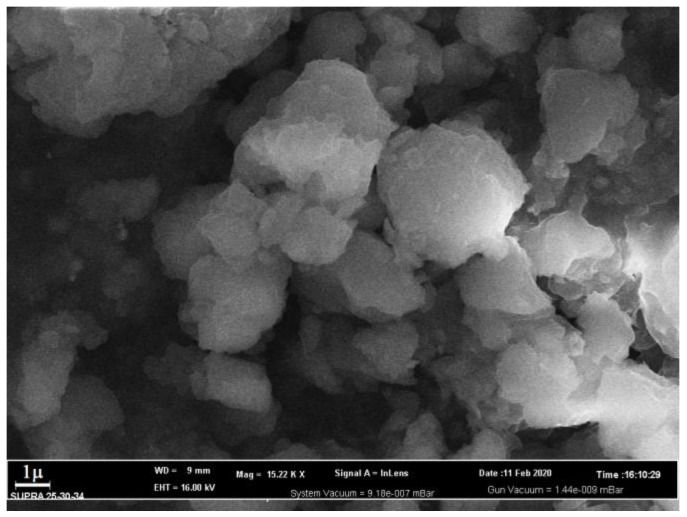
SEM image of cobalt terephthalate crystals after the antibiotic adsorption process.

After the adsorption process, a loss of clear contours of cobalt terephthalate crystals and a general blurring of the image are visually observed while maintaining the shooting parameters on the SEM image of the cobalt terephthalate sample (Figure 11).

### 3.3. Adsorption Isotherms

The data obtained during the experiment on the study of the adsorption properties of selected cephalosporin antibiotics by the studied CP were analyzed using the Langmuir and Freundlich models.

Plots of Langmuir isotherms in unlinearized and linearized forms for CZO, CTX and CRO, respectively, as well as approximation equations are obtained. Sufficiently high correlation coefficients (Table 2) show that the calculated parameters of the Langmuir model satisfactorily describe the antibiotic adsorption process. The maximum adsorption value calculated using the Langmuir model agrees satisfactorily with the experimentally obtained value.

The model of the Freundlich adsorption isotherm also agrees well with the obtained experimental data (R^2^ more than 0.89) (Table 2). The correlation coefficients indicate that the adsorption process is better described by the Freundlich model than by the Langmuir model. The obtained values of the Freundlich constants show that cobalt terephthalate is an effective sorbent for the adsorption of cephalosporin antibiotics. The coefficient 1/n vary from 0 to 1 (Table 2), indicating suitable adsorption conditions. This agrees well with other experimental data.

### 3.4. Adsorption Kinetics

To describe the process of adsorption of cephalosporin antibiotics on cobalt terephthalate, we used kinetic models of pseudo-first and pseudo-second order reactions. Analysis of plots of ln(q_e_ − q_t_) vs. t for a pseudo-first order reaction and t/q_t_ vs. t for a pseudo-first-order reaction of antibiotic adsorption made it possible to calculate adsorption rate parameters (Table 3). At the initial stages of the adsorption process, the regularities of adsorption satisfactorily describe the pseudo-first order equation. At this point, the process is significantly affected by the phenomenon of film diffusion. Subsequently, an increase in the concentration of sorbate molecules on the surface of the sorbent stimulates the movement of sorbate molecules into the pores of the sorbent under the action of a concentration gradient. Then, this process slows down and leads to the appearance of other adsorption mechanisms. The obtained correlation coefficients allow us to conclude that the pseudo-second-order kinetic model is more preferable for describing the adsorption process of cephalosporin antibiotics than the pseudo-first-order kinetic model.

### 3.5. Thermodynamics of Adsorption

The thermodynamic characteristics of the adsorption process were calculated graphically from the dependence of the thermodynamic equilibrium constant on the reciprocal temperature and are presented in Table 4. An increase in temperature leads to an increase in the Gibbs free energy, which indicates a higher adsorption efficiency at moderately low temperatures. The negative ∆G values (−20 kJ/mol < ∆G < 0 kJ/mol) for all analyzed temperatures show that the adsorption process is spontaneous and thermodynamically favorable, and adsorption of the cephalosporin antibiotics on CP has a physical nature. The exothermic nature of the adsorption process is confirmed by the negative value of ∆H^0^. A positive value of ∆S^0^ indicates the affinity of CP for adsorbed antibiotics. This suggests an increase in the randomness of the solid/solution interface during the adsorption of antibiotic molecules on the surface of the adsorbent, probably due to structural changes in both the adsorbate and the adsorbent.

### 3.6. Reusability

**Figure 12:**
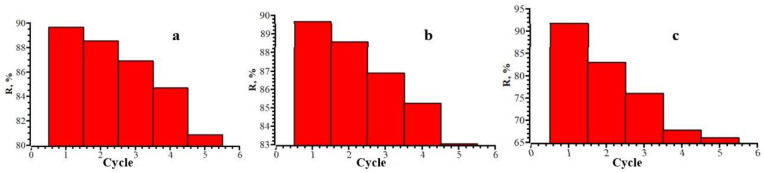
Dependence of the degree of extraction of CZO (**a**), CTX (**b**), and CRO (**c**) on the number of working cycles.

One of the most important properties of an adsorbent in terms of its economic efficiency is its reusability [70]. To determine the number of working cycles, adsorption–desorption experiments were carried out five times (Figure 12). The regenerated adsorbent still retained a high adsorption capacity after five cycles. The probable reason for the slight decrease in the adsorption capacity of the adsorbent is the irreversible occupation of partial adsorption sites.

### 3.7. Separation of a Mixture of Antibiotics on a Chromatographic Column

When separating a mixture of antibiotics, it was found that CZO was eluted first, and 10 mL of the eluate was obtained, which contained 96.7% of the antibiotic compared to that introduced into the column. Cefriaxone and cefotaxime are poorly separated on the column and were obtained as a mixture containing 93.4 and 98.5% of CRO and CTX, respectively, by weight introduced into the column. The results of the experiment are shown in Table 5.

Thus, the synthesized sorbent based on cobalt terephthalate shows a good sorption capacity compared to other known sorbents, which is shown in Table 6.

We assume that the mechanism of adsorption in this case may consist not only in the distribution of antibiotic molecules inside the pores of the sorbent held by physical forces, but also through other interactions, such as π-π stacking or the formation of hydrogen bonds between hydrogen at nitrogen atoms and carboxyl groups of the sorbent. In addition, it can be assumed that the carboxyl groups of the antibiotic are coordinated with cobalt atoms, which makes it possible to retain an additional number of sorbate molecules. We believe that such a complex of interactions largely promotes to the sorption of the antibiotic on this sorbent. This assumption correlates well with the thermodynamics of the process.

## 4. Conclusions

The coordination polymer based on cobalt terephthalate was synthesized at low temperatures. Its structure was studied by SEM, XRD, and IR spectroscopy. The dependence of the solid-phase extraction of antibiotics on the initial concentration of the antibiotic, the contact time, and pH of the medium was studied. It has been established that the complex is an effective sorbent for the extraction of cephalosporin antibiotics. The influence of temperature on the sorption of antibiotics was proved and it was concluded that the best results were obtained at low temperatures. The conducted studies have shown the possibility of reusing cobalt terephthalate as a sorbent. The use of a sorbent makes it possible to separate the studied antibiotics on a chromatographic column.

## Figures and Tables

**Table 1:** Investigated cephalosporins and their characteristics.

Analyte	Acronym	CAS No.	Molecular Structure	Molecular Weight	logK_ow_^a^	pK_a_^b^
Ceftriaxone	CRO	73384-59-5	C_18_H_18_N_8_O_7_S_3_	554.58	0.78	3, 3.2, 4.1
Cefotaxime	CTX	63527-52-6	C_16_H_17_N_5_O_7_S_2_	455.46	0.64	3.8
Cefazolin	CZO	25953-19-9	C_14_H_14_N_8_O_4_S_3_	454.51	−0.58	2.1

^a^ logK_ow_ from [49,60,61]. ^b^ pK_a_ from [1].

**Table 2:** Values of parameters of adsorption isotherms.

Model	Sorbate	Index	T, °C		
			5	23	35
Langmuir	CZO	q_max_	308.6	321.5	300.3
		K_L_	0.28	0.49	0.48
		R^2^	0.945	0.846	0.619
	CTX	q_max_	496.0	396.8	243.2
		K_L_	0.151	0.268	0.457
		R^2^	0.770	0.903	0.947
	CRO *	q_max_	520.8	429.2	458.7
		K_L_	0.062	0.056	0.067
		R^2^	0.707	0.761	0.847
Freindlich	CZO	1/n	0.55	0.57	0.55
		K_F_	2.56	2.18	1.88
		R^2^	0.992	0.974	0.899
	CTX	1/n	0.67	0.68	0.69
		K_F_	3.1	4.1	4.8
		R^2^	0.970	0.972	0.941
	CRO *	1/n	0.69	0.58	0.58
		K_F_	1.87	2.18	2.18
		R^2^	0.925	0.943	0.943

* Adsorption was carried out at 2 °C.

**Table 3:** Kinetic parameters of the adsorption process.

Sorbate	t, °C	q_e_, mg/g	R^2^		k_1_, min^−1^	k_2_, g/mg min
			Pseudo First Order	Pseudo Second Order		
CZO	5	193.10	0.823	0.877	0.8	1.61
	23	192.80	0.920	0.884	0.8	1.80
	35	191.20	0.983	0.971	0.83	1.41
CTX	5	193.10	0.936	0.993	0.8	0.68
	23	192.80	0.941	0.994	0.57	1.76
	35	191.20	0.983	0.992	0.75	1.10
CRO	2	193.10	0.881	0.882	0.975	0.56
	23	192.80	0.876	0.861	0.925	0.76
	35	191.20	0.880	0.891	0.900	0.31

**Table 4:** Thermodynamic parameters of adsorption of antibiotics.

Sorbate	T, K	K_c_	ΔG^0^, kJ/mol	ΔH^0^, kJ/mol	ΔS^0^, J/mol K
CZO	278	2.535	−2.150	−2.123	9.321
	296	2.387	−2.141		
	308	1.260	−0.563		
CTX	278	2.560	−2.173	−1.844	11.230
	296	2.387	−2.141		
	308	1.377	−0.819		
CRO	275	1.363	−0.717	−0.332	4.416
	296	1.162	−0.369		
	308	1.234	−0.538		

**Table 5:** Separation of a mixture of antibiotics.

Factor	Index
Degree of extraction from the column, %	CZO—96.7 CRO—93.4 CTX—98.5
R^2^	CZO—0.965 CRO—0.974 CTX—0.987
The degree of resolution from the mixture, arb. units	CZO—1.345 CRO—0.5 CTX—0.5
Range of measured mass concentrations, mg/L	3.25–100
q_max_, mg/g	CZO—365.2 CRO—396.8 CTX—361.1
Repeatability	0.0047
Accuracy index ± Δ, % (error characteristic)	2.7 ± 5%

**Table 6:** Comparison of the effectiveness of sorbents.

Sorbent	q_e_, mg/g	Ref.
Amberlite XAD4	211.67	[71]
Amberlite XAD16	86.75	[71]
Activated Carbon	222.08	[71]
Activated Carbon	290.1	[72]
N-Bentonit-N-TiO_2_-Chitozan	90.91	[73]
Clinoptilolite	76	[74]
Cobalt Terephthalate	361.1	this work

## Data Availability

Not applicable.
